# Probiotic, Prebiotic, and Brain Development

**DOI:** 10.3390/nu9111247

**Published:** 2017-11-14

**Authors:** Tomás Cerdó, Alicia Ruíz, Antonio Suárez, Cristina Campoy

**Affiliations:** 1 Department of Paediatrics, School of Medicine, University of Granada, 18016 Granada, Spain; tcr@ugr.es; 2 EURISTIKOS Excellence Centre for Paediatric Research, Biomedical Research Centre, University of Granada, 18016 Granada, Spain; aliruizrodriguez@ugr.es; 3 Department of Biochemistry and Molecular Biology 2, Biomedical Research Centre, University of Granada, 18016 Granada, Spain; asuarez@ugr.es; 4 Spanish Network of Biomedical Research in Epidemiology and Public Health (CIBERESP), Carlos III Institute, 18016 Granada, Spain; 5 Department of Paediatrics, Faculty of Medicine, University of Granada, Av. de la Investigación, 11, 18016 Granada, Spain

**Keywords:** microbiota, prebiotics, probiotics, health, disease

## Abstract

Recently, a number of studies have demonstrated the existence of a link between the emotional and cognitive centres of the brain and peripheral functions through the bi-directional interaction between the central nervous system and the enteric nervous system. Therefore, the use of bacteria as therapeutics has attracted much interest. Recent research has found that there are a variety of mechanisms by which bacteria can signal to the brain and influence several processes in relation to neurotransmission, neurogenesis, and behaviour. Data derived from both in vitro experiments and in vivo clinical trials have supported some of these new health implications. While recent molecular advancement has provided strong indications to support and justify the role of the gut microbiota on the gut–brain axis, it is still not clear whether manipulations through probiotics and prebiotics administration could be beneficial in the treatment of neurological problems. The understanding of the gut microbiota and its activities is essential for the generation of future personalized healthcare strategies. Here, we explore and summarize the potential beneficial effects of probiotics and prebiotics in the neurodevelopmental process and in the prevention and treatment of certain neurological human diseases, highlighting current and future perspectives in this topic.

## 1. Introduction

The micro-organisms that inhabit the human gastrointestinal tract (GI) have been implicated in the development and functioning of a number of basic physiological processes, such as digestion, immunity, and the maintenance of homeostasis. The GI microbiota may also play a role in multiple diseases, ranging from inflammation to obesity [1,2]. Recently, many studies have shown that gut microbiota play a very important role in the development and function of the central nervous system (CNS) through specific channels, such as metabolic, neuroendocrine, and immune pathways [3]. In particular, these researchers have found bi-directional communication between the brain and the gut microbiota, denominated the microbiota–gut–brain axis [4,5,6].

Although the molecular mechanisms by which the gut microbiota communicate with the brain are not yet clear, the link between both components is currently attributed to immune signals and the vagus nerve. Cellular components produced by gut microbiota, such as lipopolysaccharide (LPS), peptidoglycan, and flagellin, are recognized by pattern-recognition receptors (PRRs), such as Toll-like receptors (TLRs), NOD-like receptors (NLRs), or RIG-1-like receptors (RLRs), on epithelial and immune cells, producing cytokines, hormones, and other molecular signals, which will act as neurotransmitters within the CNS [7]. Several studies have found that, in the densely innervated gut, the vagus nerve is involved in the bi-directional communication of the microbiota–gut–brain axis [8,9], while others have shown vagus-independent effects [10,11]. Either way, a supplementing nutrition therapy with specific probiotic commensals and prebiotics can alter the excitability of enteric nervous system (ENS) sensory neurons [12,13,14]. Prebiotics-induced growth of probiotic members within the *Bifidobacterium* and *Lactobacillus* genera show multiple beneficial effects on host immunity and physiology [15]. Moreover, strong effects of *Bifidobacterium* and *Lactobacillus* spp. on the brain–gut axis have been reported [16].

This review summarizes current knowledge on the influence of the establishment of the gut microbiota in critical neurodevelopmental windows, and discusses recent findings on the interactions between the gut microbiota and the host’s brain–gut axis communications. In addition, current research on the effects of the administration of probiotics and prebiotics in specific neurological disorders is reviewed. Finally, recommendations for future research on this topic are also discussed.

## 2. Establishment of Intestinal Microbiota during Early Neurodevelopmental Windows

Gut microbiota establish a beneficial cohabitation with the host that will prime for health later in life [17]. The assembly of the gut microbiota occurs during the first three years of life, starting from birth, where there is a rapid rate of colonization and expansion of gut bacteria dominated by *Actinobacteria* and *Proteobacteria* that shifts towards one dominated by *Firmicutes* and *Bacteroidetes*, increasing compositional diversity and stability while maturing into an adult-like state [18]. This process coincides in time with the intense synaptogenesis and pruning in the cerebral cortex during early life [18,19,20], ending in adolescence [21]. Therefore, perturbations of gut microbiota colonization and maturation by environmental factors may influence brain development. The dynamics of the microbial ecosystem’s maturation during this critical period of CNS development is influenced by several environmental factors, such as mother-to-child bacterial transfer, mode of delivery, and type of feeding. The mother-to-child transfer of commensal bacteria in the uterus has been shown to influence an infant’s immune system development [22,23]. Until recently, the idea that foetuses were sterile in the uterus and that the microbial colonization of the new-born started during and after birth had been widely accepted [24]. However, nowadays, this belief has been challenged by evidence of microbes in placenta and other tissues surrounding the foetus, such as umbilical cord blood after vaginal and caesarean birth [25,26,27]. Several studies have analysed the meconium of new-born babies and showed the presence of bacterial populations, including *Enterococcus*, *Lactococcus*, *Escherichia*, *Leuconostoc*, and *Streptococcus*, though at low levels, concluding that gut colonization occurs mainly after birth [28]. Based on these findings, prenatal probiotic intervention has been shown to modulate the expression of TLR-related genes in the placenta and foetal GI tract and to reduce atopic dermatitis [29,30]. Thus, prenatal and postnatal maternal oral probiotic therapy may represent an effective method of intervention to prevent pathologies such as allergy [31], atopic diseases [32], and neurodevelopmental disorders, reviewed below. Still, the origin of the microbiota colonizing the placenta is unknown and results have to be carefully interpreted, because, in samples with low microbial biomass, such as those from placenta, the risk of contamination is high when using high-throughput sequencing methods based on DNA amplification [29]. Further studies are needed to discern whether foetuses have contact with bacteria before birth or are colonized during and after parturition.

Regardless of mother-to-child transmission within the intrauterine environment, two different modes of maternal–infant transmission during delivery have been proposed: (a) horizontal, in which microbes are taken up from the environment for infants born by caesarean section; and (b) vertical, in which vaginal microbes are transferred during parturition to the infants [33]. Infants delivered by Caesarean section are more likely to suffer several diseases, such as asthma, obesity, or allergies, in adulthood [34]. Interestingly, a study carried out by Jasarevic et al. using a mouse model of early prenatal stress found that changes in the vaginal microbiome were associated with shifts in the abundance of *Lactobacillus* in the expression of maternal stress proteins related to vaginal immunity, in offspring metabolic profiles related to energy balance, and in the amino acid profiles of the developing brain [35].

The third strong environmental factor that influences an infant’s gut microbial development as well as neurodevelopment is the type of feeding. In recent years, several studies have reported that breastfeeding and particularly full breastfeeding has beneficial effects on child neuropsychological development [36]. Human milk is the optimal feeding source, since it provides all the nutrition factors that an infant needs for healthy development. Human milk is not sterile, and, during breastfeeding, bacteria from mother’s skin and mammary gland via maternal dendritic cells and macrophages [37] are transferred to the baby [38]. Breast-fed infants tend to contain a more uniform population of gut microbes dominated by *Bifidobacterium* and *Lactobacillus* [39], whereas formula-fed infants exhibit higher proportions of *Bacteroides*, *Clostridium*, *Streptococcus*, *Enterobacteria*, and *Veillonella* spp. [21]. Bacteria belonging to the *Bifidobacterium* genus present in human milk are early colonizers that characterize the gut microbial composition of healthy breast-fed new-born’s [40] with beneficial functions for the host, such as the acceleration of the maturation of the immune response, the limitation of excessive inflammation, the improvement of the intestinal permeability, and an increase of acetate production [41]. In mice, *B. infantis* produces antidepressant-like effects and normalizes peripheral pro-inflammatory cytokine and tryptophan concentrations, both of which have been implicated in depression [42,43,44]. Moreover, breastfeeding has an additional role in the establishment of an infant’s gut microbiota, since it contains bioactive molecules that are increasingly recognized as drivers of microbiota development and overall gut health [45]. Among the nutrients present in human milk, oligosaccharides constitute the third-most abundant class of molecules in terms of concentration after lactose and lipids. Nowadays, more than 200 different structures have been identified as human milk oligosaccharides (HMOs) [35]. HMOs can act as prebiotics, stimulating the growth of specific bacterial groups such as *Staphylococci* [46] and *Bifidobacteria* [47].

These results suggest that postnatal neurodevelopment and gut microbiota establishment co-occur, suggesting the intriguing possibility of a bi-directional regulation of each other’s maturation [48]. Further studies are needed in order to clarify whether those differences in bacterial acquisition during early life lead to neurodevelopmental differences in infants.

## 3. Gut Microbiota–Brain Axis

**Figure 1:**
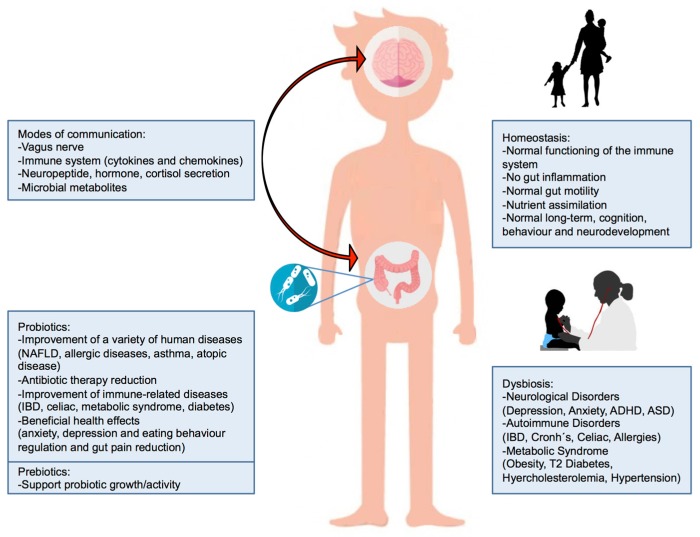
The gut microbiota–brain axis. The central part of the figure shows the bidirectional influence between the brain and gut microbiota. The left side of this figure shows modes of communication in the bidirectional crosstalk between gut microbiota and the brain and the possible influences of prebiotics and probiotics on human diseases. The right side of the figure shows the consequences of gut dysbiosis/homeostasis. Intestinal dysbiosis can adversely influence gut physiology, leading to inappropriate brain–gut axis signalling and associated consequences for CNS functions and disease states. Abbreviations: Non-Alcoholic Fatty Liver Disease (NAFLD), Inflammatory Bowel Disease (IBD), Attention deficit hyperactivity disorder (ADHD), Autism spectrum disorder (ASD).

The brain and the gut reciprocally influence each other by constant communication (Figure 1). The brain–gut–microbiota axis includes the CNS, the endocrine-immune system, the hypothalamus–pituitary–adrenal (HPA) axis, the autonomic nervous system, the ENS, and the gut microbiota [49]. This bi-directional communication enables signalling from the brain to influence motor, sensory, and secretory modalities of the GI tract, and conversely, signalling from the gut to affect brain function, most notably the hypothalamus and amygdala that are implicated in stress [50,51,52].

Though communication between brain and gut was realized in the middle of the nineteenth century [53], gut microorganisms had not been considered important for the development and function of the CNS or for brain diseases until recently, expanding the term to microbiome–gut–brain axis [54]. In humans, evidence of microbiome–gut–brain axis interactions have been obtained from the association of shifts in gut microbiota composition with central nervous disorders (i.e., autism spectrum disorder (ASD) and anxiety and depressive behaviours) and functional gastrointestinal disorders [54]. Most of the data demonstrating the role of the microbiota in the gut–brain axis have been obtained from germ-free animals [55]. Mice fed with prebiotics showed diminished stressor-induced anxiety-like behavior [56]. In a mouse model of ASD, Buffington et al. showed that a maternal high-fat diet reduced the number of oxytocin immunoreactive neurons in the hypothalamus and induced dysbiosis that was restored by a commensal *Lactobacillus* *reuteri* strain [57]. In a mouse model of Parkinson’s disease, Sampson et al. highlighted a negative interaction in the microbiome–gut–brain axis because the absence of gut bacteria decreased aggregated misfolded α-synuclein levels and reduced the severity of the animals’ abnormal movements. The authors showed that short chain fatty acids (SCFA), such as acetate, propionate, and butyrate, the end products of anaerobic fermentation of dietary fibre and starch, promoted a microglia-mediated immune response and increased α-synuclein aggregation, causing movement abnormalities [58]. Butyrate can cross the blood-brain barrier (BBB) and produce a dose-dependent increase in neuronal and glial nuclear histone H3 acetylation in mice due to its potential to inhibit histone deacetylation [59]. Another metabolite whose levels in the host are influenced by gut microbiota is tryptophan, the amino acid precursor of the neurotransmitter serotonin, and kynurenine, the main breakdown product of tryptophan catabolism [60]. Kynurenine intake during gestation and postnatal development, a time frame in which the maternal and offspring microbiota undergo major compositional and functional remodelling, produced neurochemical and cognitive deficits later in adulthood [61]. The prenatal inhibition of kynurenine synthesis modified hippocampal neuron morphology and changed neocortical and cerebellar protein expression that persisted into adulthood. In germ-free and in antibiotic-induced microbiota-depleted mice, despite increased circulating tryptophan levels, serotonin and kynurenin availabilities were decreased, suggesting that gut microbiota modulated kynurenin metabolism [62]. Distinct gut microbial species affect host physiology, producing diverse neuromolecules involved in mood regulation. *Lactobacillus* and *Bifidobacterium* spp. generate gamma-aminobutyric acid (GABA). *Candida*, *Streptococcus*, *Escherichia*, and *Enterococcus* spp. synthesise serotonin while *Bacillus* spp. produces dopamine [63].

Gut microbiota also influence the regulation of BBB integrity. The BBB is an active interface between systemic circulation and the CNS that maintains brain homeostasis by preventing the entry of potentially toxic or harmful substances and regulates the transport of nutrients and the removal of metabolites [64]. Braniste et al. (2014) [65] showed that the transplantation of gut microbiota into germ-free mice normalized BBB permeability and upregulated the expression of tight junction proteins. Therefore, gut microbiota have a key role in regulating BBB permeability, suggesting that the maternal gut microbiome influences an offspring’s BBB integrity. Together with the results discussed in the previous section, these findings open an intriguing question on the mechanism by which a mother’s gut microbiota cooperate in regulating BBB integrity and ultimately brain function development.

Gut microbiota have direct effects on the immune system, which constitutes another route of communication between gut microbes and the brain. The signalling molecules of the immune system, cytokines and chemokines, access the brain from the periphery via the vagus nerve or directly via the circumventricular organs [66]. The administration of rifaximin (a non-systemic, broad-spectrum antibiotic) to stressed rats increased the abundance of *Lactobacillus* in the ileum and the expression of the tight junction protein occludin while decreasing the expression of pro-inflammatory interleukin 17, interleukin 6, and tumour necrosis factor α mRNA [67].

Since many of the above effects have been observed during early life, it is plausible that an environmentally induced dysbiosis of infants’ microbiota (e.g., mode of birth, maternal transmission of a suboptimal microbiota, antibiotics) may generate altered patterns of microbial metabolites with detrimental effects in human CNS development. Further research is needed to unravel these mechanisms and develop probiotics or prebiotics therapies that shape gut microbial composition and metabolism to ultimately modulate CNS development.

## 4. Probiotics

In 2001, the Food and Agriculture Organization of the United Nations and the World Health Organization (FAO/WHO) proposed the following definition of probiotics: “live micro-organisms which, when administered in adequate amounts, confer a health benefit on the host” [68], which was reaffirmed in 2014 [69]. Probiotics, comprised by strains of *Lactobacilli*, *Bifidobacteria* and *Saccharomycetes* have been suggested to play a role in fighting human diseases, such as non-alcoholic fatty liver disease (NAFLD), allergy diseases, and asthma. They also promote protection against atopic disease in the infant during pregnancy and breastfeeding [32,70,71]. In addition, probiotics also reduce the duration of antibiotic therapy, and reduce symptom severity in immune-related diseases, such as inflammatory bowel diseases (IBDs), celiac disease, metabolic syndrome and diabetes [72,73].

The search for probiotics that can affect cognitive functions, known as psychobiotics, has increased in recent years (Table 1). Psychobiotics are defined as live organisms that, when ingested in adequate amounts, produce beneficial health effects to patients suffering from psychiatric illness [74]. Depression is currently a major psychiatric disorder in developed countries, and is characterized by a low mood or loss of interest and anxiety affecting appetite and sleep. Messaoudi et al. [75,76] reported a double-blind, placebo-controlled, randomized study where a multispecies probiotic containing *Lactobacillus helveticus* R0052 and *Bifidobacterium longum* R0175 (PF) was administered to healthy women for 30 days. This treatment resulted in a decrease in the global scores of the hospital anxiety and depression scale (HADs) and the global severity index of the Hopkins symptoms checklist (HSCL-90) due to the decrease of the sub-scores of somatization, depression, and anger–hostility spheres. In a cohort of 124 healthy humans, Benton et al. reported that the consumption of *Lactobacillus* *casei*-containing yogurt improved the self-reported mood of those whose mood was initially poor [77]. Similarly, Steenbergen et al. reported a significantly reduced overall cognitive reactivity to depression, in particular aggressive and ruminative thoughts, in forty healthy young adults that consumed either a probiotic supplement or placebo for 4 weeks [78]. Recently, Akkasheh et al. showed that the consumption of a probiotic supplement significantly decreased Beck Depression Inventory (BDI) scores, indicating overall improved symptoms, including mood, in 40 patients diagnosed with depression [79]. Conversely, Marcos et al. reported that probiotics decreased, respectively, levels of stress and anxiety assessed using the state-trait anxiety inventory (STAI) that remained unchanged in subjects under academic examination stress [80]. In a recent study carried out by Romijn et al. [81], administering a multispecies probiotic containing *L. helveticus* and *B. longum* in 79 participants that were not taking psychotropic medications at that moment and with at least moderate scores on self-report mood measures, found no evidence that the probiotic formulation was effective in treating low mood or in moderating the levels of inflammatory and other biomarkers. Improved cognitive function (neuropsychological and cognitive fatigue) was reported by Chung et al., which tested a *L. helveticus*-fermented milk in healthy 60–75 year olds, though no effects on stress or geriatric depression symptoms were observed [82].

Probiotics affect mood by their ability to modulate pain in the gut. A recent study reported that the administration of *Lactobacillus reuteri* DSM 17938 in the treatment of children with functional abdominal pain (FAP) and irritable bowel syndrome (IBS) is associated with a possible reduction of the intensity of pain [83]. In 35 patients suffering from chronic fatigue syndrome, Rao et al. showed that while the consumption of the probiotic improved anxiety scores, it had no effect on depressive symptoms [84]. Giannetti et al. also reported that a probiotic mixture of *B. infantis* M-63, *B*. *breve* M-16V, and *B longum* BB536 was associated with improvement in children with IBS, but not in children with functional dyspepsia (FD) [85]. In healthy women without gastrointestinal or psychiatric symptoms, the consumption of a fermented milk product containing *B. animalis* subsp. *lactis*, *Streptococcus thermophilus*, *Lactobacillus bulgaricus*, and *L. lactis* subsp. *lactis* resulted in robust alterations in activity in the brain regions that control the central processing of emotions and sensations, as observed by functional magnetic resonance imaging [86].

Probiotics have been tested to normalize gut microbial composition and metabolism, enhance gut barrier, and relieve patients suffering from ASD. In 2012, Kaluzna-Czaplinska and Blaszczyk reported that the administration of *Lactobacillus acidophilus* in 22 ASD subjects decreased d-arabinitol concentration and the ratio of d-arabinitol to l-arabinitol in urine, and improved their ability to follow directions, as demonstrated through a comparison with data collected before the treatment [87]. Another study reported that a combination of *Lactobacillus acidophilus*, *L. casei*, *Lactobacillus delbrueckii*, *B. longum* and *Bifidobacterium bifidum*, formulated with the imunomodulator Del-Immune V (*Lactobacillus rhamnosus* V lysate), decreased the severity of ASD symptoms and improved GI symptoms in 33 children [88]. Moreover, a recent study of “Children Dophilus” (a combination of three species of *Lactobacillus*, two species of *Bifidobacterium* and one strain of *Streptococcus*) in 10 ASD children showed higher GI dysfunction in ASD children and siblings and a very strong association of the amount of *Desulfovibrio* spp. with the severity of autism. After the intervention, the *Bacteroidetes*/*Firmicutes* ratio, *Desulfovibrio* spp., and the amount of *Bifidobacterium* spp. were normalized in faeces of autistic children [89]. However, the effects of treatments with probiotics on children with ASD need to be evaluated through rigorous, controlled trials. In a recent clinical study currently in progress, Santocchi et al. are providing a multispecies probiotic (one strain of *S. thermophilus* DSM 24731, three strains of *Bifidobacterium* (*B. breve* DSM 24732, *B. longum* DSM 24736, and *B. infantis* DSM 24737), and four strains of *Lactobacillus* (*L. acidophilus* DSM 24735, *Lactobacillus plantarum* DSM 24730, *Lactobacillus paracasei* DSM 24733, and *L. delbrueckii* subsp. *bulgaricus* DSM 24734) to a group of 100 pre-schoolers with ASD. This study will try to provide new insights to clinical and neurophysiological patterns in response to a probiotic mixture in ASD patients [90].

Probiotics are also tested in the treatment of schizophrenia and bipolar disorder. One of the first trials of probiotic compounds in schizophrenia used a combined probiotic of *L. rhammosus* strain *GG* and *B. animals* subsp. *Lactis strain* Bb12. The results showed no significant difference in psychiatric symptom severity between probiotic and placebo supplementation [91]. However, other studies have found that probiotic supplementation significantly alters the levels of several serum proteins, including the von Willebrand factor and the brain-derived neurotrophic factor, and lowered the level of antibodies to the fungus *Candida albicans* [92,93].

Despite that the majority of the studies found positive results on symptoms in these neurological disorders, future studies are needed to identify potential probiotics for the effective modulation of these disorders as well as to define probiotics risk in therapeutic interventions. Gut microbial studies that use 16S rRNA gene sequencing to characterize bacteria must consider that highly similar bacteria (higher than 97% sequence identity) can have large differences in genomic sequences and profound differences in growth and metabolism. Hence, it is important to characterize probiotics to the strain level and apply next-generation sequencing techniques to analyse the functions encoded by their genome [94]. Therefore, the effects of one probiotic strain should not be generalized to others without confirmation in separate studies.

## 5. Prebiotics

Although the concept of a prebiotic was first defined in 1995 by Gibson, the current definition of a prebiotic is the one proposed by the International Scientific Association for Probiotics and Prebiotics (ISAPP): a substrate that is selectively utilized by host micro-organisms and confers a health benefit [95]. The group of substances recognized for their ability to influence gastrointestinal health comprise certain non-digestible oligosaccharides (NDOs), soluble fermentable fibres, and HMOs. NDOs are low molecular weight carbohydrates in nature that are intermediates between simple sugars and polysaccharides. The use of NDOs as prebiotics has rapidly increased because the enrichment of a diet with NDOs provides the opportunity to improve the gut microbial ecosystem, including bacterial populations, biochemical profiles, and physiological effects [96]. Fibre influences satiety by the following two mechanisms. One is by increasing the chewing time of fibre-rich foods, which promotes saliva and gastric acid production and increases gastric distension, triggering afferent vagal signals of fullness contributing to this end. The other mechanism is by slowing gastric emptying and decreasing the rate of glucose absorption in the small intestine. Consequently, the insulin response may also be attenuated; this is sometimes correlated with satiation and satiety [97]. Various hormones (i.e., ghrelin, the polypeptide YY, and the glucagon-like peptide) have been related to satiety, and are sent to the brain, where they regulate food intake and overall energy balance [98].

Though prebiotic therapies potentially could be beneficial for children with a genetic pre-disposition to develop ASD or attention deficit hyperactivity disorder because of their selective enhancement of *Lactobacilli* and *Bifidobacteria* growth [99], a small number of studies has examined the effect of these prebiotics on disorders related to CNS (Table 2). Inductive evidence that prebiotics modulated emotional satisfaction was provided by Hume et al., who investigated the effect of oligofructose-enriched inulin/d administration versus a placebo (maltodextrin) in a randomized, double-blind, placebo-controlled trial with 42 children (who were aged 7–12 and were overweight and obese) [100]. Prebiotic supplementation improved subjective appetite ratings, reducing energy intake in older but not in younger children.

In a cohort of healthy male and female subjects (*n* = 45), Schmidt et al. tested the intake of fructo-oligosaccharides (FOS) and Bimuno^®^-galactooligosaccharides (B-GOS), and reported that only B-GOS reduced the waking-cortisol response [101]. Exaggerated waking cortisol is a biomarker of emotional disturbances, such as depression [102]. Besides this, the subjects also provided measures of vigilance, or attention to negative stimuli, which is also a behavioral marker of anxiety and depression [103]. B-GOS attenuated vigilance, suggesting a reduction in anxiety and depression [104]. Van den berg et al. found no evidence that the use of short-chain galacto-oligosaccharides/long-chain fructo-oligosaccharides/pectin-derived acidic oligosaccharides in preterm infants at 24 months improves neurodevelopmental outcomes [105]. LeCouffe et al. studied the effect of an enteral supplementation of a prebiotic mixture (neutral and acidic oligosaccharides) in the neonatal period and found no effect on neurodevelopment [106], though lower *Bifidobacteria* counts are associated with serious neonatal infections and lower neurodevelopmental outcomes.

More studies are required to determine whether prebiotics exert a beneficial effect on neurodevelopmental disorders in infants, and to understand the mechanism of action, by stimulating certain bacterial taxa or bacterial activities within gut microbiota. Efficacy, safety, and dosing schedules should be established for each prebiotic product in long-term follow-up studies.

## 6. Synbiotics

The term synbiotic was primarily stated considering the benefits of a product that combines prebiotics and probiotics and in which the prebiotic compounds selectively favour the probiotic strains [109]. Several studies have shown positive synergistic effects for synbiotics on obesity, diabetes, non-alcoholic fatty liver disease, necrotizing enterocolitis in very low birth weight infants, and in the treatment of hepatic encephalopathy [110,111,112,113,114]. Despite these findings, few studies have tested the potential benefits of synbiotics on neurodevelopmental disorders (Table 2). Malaguarnera et al. reported that *B. longum* plus FOS improved cognitive function in the treatment of minimal hepatic encephalopathy (MHE) [107]. Firmansyah et al. provided milk containing synbiotics (BL999, LPR, and prebiotics) and LCPUFA to 393 healthy toddlers at 12 months-old for 12 months. The authors reported that the change in cognitive and adaptive behaviour scores between 12 and 16 months was higher but not significantly different in the synbiotics group compared with the control group [108]. Future work is needed to determine whether synbiotics may contribute to relieve neurological diseases and to explore the benefits of new potential synbiotics during critical time windows in an infant’s CNS development and susceptibility to neurological disorders.

## 7. Future Perspectives

During the last decade, numerous in vivo and in vitro studies have explored the influence of probiotics and prebiotics in host physiology [115]. Their results showed that gut microbiota may modulate inflammation, adiposity, satiety, energy expenditure, and glucose metabolism. Most efforts have focused on studying the mechanisms by which certain probiotics regulate the colonization of and protect against pathogens through the activation of the mucosal immune system and competition for limited nutrients [116,117]. Alternate approaches such as recombinant probiotics expressing therapeutic biomolecules, faecal microbiota transplantation and phage therapy, need be explored for the manipulation of the gut ecosystem. A proof of concept was the experiment performed by Paton et al., where they created a recombinant probiotic by introducing glycosyltransferase genes from *Neisseria meningitidis* or *Campylobacter jejuni* in a harmless *Escherichia coli* strain (CWG308) to treat and prevent the diarrheal disease caused by enterotoxigenic *E. coli* strains [118]. The same group also developed a recombinant probiotic for the treatment and prevention of cholera [119]. A recent study showed that microbiota transfer therapy improves ASD symptoms in children, which persists for at least 8 weeks after the treatment ends [120]. And finally, phage therapy has become an interesting strategy to treat bacterial infections due to the rise of antibiotic­resistant microbial strains. The only approved phage therapy clinical trial in the human gut was carried out in 120 patients with diarrhoea caused by *E. coli*, who were infected by a coliphage mix. The treatment failed to solve diarrhoea, although no adverse effects of phage infection were observed [121]. Customized phage cocktails could be an alternative for future therapies. These phages would directly target pre-identified bacterial pathogens though the main drawback would be the high inter­individual variation of the gut microbiome and legislative approval [122,123].

In conclusion, this review summarized the accumulating evidence on the modulation of gut microbial composition and metabolism as a potential strategy for neurological disorders and CNS development. Despite this wealth of information, the effect of probiotics and prebiotics is still largely unexplored, and numerous gaps and inconsistencies exist when the studies are compared. Differences in quantity of dose, type of strain, type of prebiotic, assessment of gut microbiota, duration of intervention, standardization of neurological measurements, variety and complexity of neurological symptoms, study design, and cohort size make it difficult to confirm evidence of efficacy. To this end, double-blind placebo in vivo studies that exploit the power of the latest robust high-throughput multi-omic technologies are required to identify the molecular mechanisms of the gut’s microbial modulation of neurological disorders and CNS development and ultimately to design effective probiotic and prebiotic therapies.

## Figures and Tables

**Table 1:** Studies evaluating probiotics supplementation on central nervous system (CNS) disorders.

Study (Reference)	Cohort Population	Probiotic Used	Key Findings
Messaoudi et al. (2011) [75,76]	55 healthy human volunteers plus 25 subjects with urinary free cortisol (UFC) levels less than 50 ng/mL (less stressed subjects), 10 subjects received the probiotic and 15 placebo.	*Lactobacillus helveticus* R0052 and *Bifidobacterium longum* R0175 (PF)	Beneficial effects on anxiety and depression related behaviors in healthy human volunteers and volunteers with lower levels of cortisol
Benton et al. (2007) [77]	124 healthy adults volunteers were randomly allocated to a group that consumed, on a daily basis, a probiotic-containing milk drink or a placebo	*Lactobacillus casei* Shirota	The consumption of a probiotic-containing yoghurt improved the mood of those whose mood was initially poor. However, there was not an increased frequency of defaecation.
Steenbergen et al. (2015) [78]	40 healthy young adults were randomly assigned to receive a 4-week intervention of either placebo or multispecies probiotics in a triple-blind intervention assessment design.	*Bifidobacterium bifidum* W23, *Bifidobacterium lactis* W52, *Lactobacillus acidophilus* W37, *Lactobacillus brevis* W63, *Lactobacillus casei* W56, *Lactobacillus salivarius* W24, and *Lactococcus lactis* (W19 and W58)	Participants who received multispecies probiotics showed a significantly reduced overall cognitive reactivity to sad mood, which was largely accounted for by reduced rumination and aggressive thoughts.
Akkasheh et al. (2016) [79]	40 patients with a diagnosis of major depressive disorder (MDD) whose age ranged between 20 and 55 years were randomized.	*Lactobacillus acidophilus*, *Lactobacillus casei*, and *Bifidobacterium bifidum.*	Patients who received probiotic supplements had significantly decreased Beck Depression Inventory total scores
Marcos et al. (2004) [80]	136 university students were randomized.	*Lactobacillus delbrueckii* subsp. *bulgaricus* and *Streptococcus salivarius* subsp. *thermophilus* plus *Lactobacillus casei* DN-114001	There was no significant treatment effect on anxiety.
Romijn et al. (2017) [81]	79 participants not currently taking psychotropic medications with at least moderate scores on self-report mood measures. Participants were randomly allocated to receive a probiotic preparation or placebo.	*Lactobacillus helveticus* and *Bifidobacterium longum*	No significant difference was found between the probiotic and placebo groups on any psychological outcome measured.
Jadrešin et al. (2017) [83]	55 children with age between 4 and 18 years old, diagnosed as functional abdominal pain (FAP) or irritable bowel syndrome (IBS) were randomly allocated.	*Lactobacillus reuteri* DSM 17938	Administration of *L. reuteri* DSM 17938 was associated with a possible reduction of the intensity of pain and significantly more days without pain in children with FAP and IBS
Giannetti et al. (2016) [85]	48 children with IBS aged between 8 and 17.9 years and 25 with functional dyspepsia (FD) with age between 8 and 16.6 years were randomized.	*Bifidobacterium infantis* M-63, *breve* M-16V, and *longum* BB536	In children with IBS a mixture of *Bifidobacteria* is associated with improvement in abdominal pain (AP) and quality of life (QoL).
Kałużna-Czaplińska et al. (2012) [87]	22 autistic children.	*Lactobacillus acidophilus*	The probiotic supplementation let to a significant decrease in d-arabinitol (DA) and the ratio of d-/l-arabinitol (DA/LA) and to a significant improvement in ability of concentration and carrying out orders
West et al. (2013) [88]	33 ASD children.	Delpro^®^ (*Lactocillus acidophilus*, *Lactobacillus casei*, *Lactobacillus delbruecki*, *Bifidobacteria longum*, *Bifidobacteria bifidum*)	88% reported a decrease in total autism treatment evaluation checklist (ATEC) score, an improvement of ASD symptoms. Participants also had significant improvements in all ATEC domains (speech/language/communication, sociability, sensory/cognitive awareness, and health/physical/behavior)
Tomova et al. (2015) [89]	10 children with autism, 9 siblings and 10 healthy children.	“Children Dophilus” containing three strains of *Lactobacillus* (60%), two strains of *Bifidobacterium* (25%), and one strain of *Streptococcus* (15%)	Probiotic diet supplementation normalized the *Bacteroidetes*/*Firmicutes* ratio, *Desulfovibrio* spp. and the amount of *Bifidobacterium* spp. in feces of autistic children. No significant difference was found to reduce symptom severity in patients with autism.
Santocchi et al. (2016) [90]	100 preschoolers with ASD on the basis of a symptom severity index specific to gastrointestinal (GI) disorders. Patients with and without GI disorders were blind randomized to regular diet with probiotics or with placebo	“Vivomixx^®^” (one strain of *Streptococcus thermophilus* DSM 24731, three strains of *Bifidobacterium* (*Bifidobacterium breve* DSM 24732, *B. longum* DSM 24736, *B. infantis* DSM 24737), and four strains of *Lactobacillus* (*Lactobacillus acidophilus* DSM 24735, *Lactobacillus plantarum* DSM 24730, *Lactobacillus paracasei* DSM 24733, *Lactobacillus delbrueckii* subsp. *bulgaricus* DSM 24734))	Ongoing study
Dickerson et al. (2014) [91] and Tomasik et al. (2015) [92]	32 patients healthy and 33 patients with schizophrenia meeting DSM-IV criteria and with at least moderately severe psychotic symptoms	*Lactobacillus rhamnosus* strain GG and *Bifidobacterium animalis* subsp. *lactis* strain Bb12	No significant difference was found to reduce symptom severity in patients with schizophrenia. Probiotic regulate immune and intestinal epithelial cells through the IL-17 family of cytokines

**Table 2:** Studies evaluating prebiotics and synbiotics supplementation on CNS disorders.

Study (Reference)	Cohort Population	Prebiotic Used	Key Findings
Prebiotics			
Hume et al. (2017) [100]	42 boys and girls, ages 7–12 years, with a body mass index (BMI) of ≥85th percentile	Oligofructose-enriched inulin/d	Prebiotic supplementation in children with overweight and obesity significantly increased feelings of fullness and reduced prospective food consumption in older but not in younger children
Schmidt et al. (2105) [101]	45 adults healthy volunteers	FOS and Bimuno^®^-galactooligosaccharides, B-GOS	B-GOS reduced waking-cortisol response and decreased attentional vigilance to negative versus positive information
van den Berg et al. (2016) [105]	77 preterm infants (gestational age <32 weeks and/or birth weight <1500 g), admitted to the level-III neonatal intensive care unit (NICU)	scGOS/lcFOS/pAOS	Neurodevelopmental outcomes were not different in the scGOS/lcFOS/pAOS and placebo group. Infections, lower bifidobacteria counts, and higher serum cytokine levels during the neonatal period were associated with lower neurodevelopmental outcomes at 24 months of age
LeCouffe et al. (2014) [106]	93 Infants, with a gestational age (GA) of less than 32 weeks and/or birth weight of less than 1500 g, participed in the study (prebiotic mixture group (*n* = 48) and placebo group (*n* = 45)	80% scGOS/lcFOS and 20% pAO	Short-term enteral supplementation of a prebiotic mixture in the neonatal period had no effect on neurodevelopmental outcome in preterm infants in the first year of life
Synbiotics			
Malaguarnera et al. (2007) [107]	60 cirrhotic patients (30 with synbiotics and 30 with placebo)	*Bifidobacterium longum* plus fructo-oligosaccharides	Patients with minimal hepatic encephalopathy (MHE) treated with *Bifidobacterium* + FOS, showed an improvement and a recovery of neuropsychological activities related to short-term memory, attention and computing ability, language, orientation ability, and cognitive activities
Firmansyah et al. (2011) [108]	393 healthy 12 month-old toddlers	The probiotic *Bifidobacterium longum* BL999 (ATCC: BAA 999) and *Lactobacillus rhamonosus*, LPR (CGMCC 1.3724), the prebiotics inulin (30%) and fructo-oligosaccharide (70%), and the LCPUFA, arachidonic acid (AA) and docosahexaenoic acid (DHA)	Changes in cognitive and adaptive behaviour scores between 12 and 16 months were higher but not significantly different in the synbiotics group compared with the control group
